# Compression of a Stearic Acid Surfactant Layer on Water Investigated by Ambient Pressure X-ray Photoelectron Spectroscopy

**DOI:** 10.1021/acs.jpcb.4c00451

**Published:** 2024-04-05

**Authors:** Harmen Hoek, Timm Gerber, Clemens Richter, Rémi Dupuy, Rebecca J. Rapf, Holger Oertel, Tillmann Buttersack, Lena Trotochaud, Osman Karslıoğlu, Dana Goodacre, Monika Blum, Sabrina M. Gericke, Christin Buechner, Bruce Rude, Frieder Mugele, Kevin R. Wilson, Hendrik Bluhm

**Affiliations:** † Chemical Sciences Division, Lawrence Berkeley National Laboratory, Berkeley, California 94720, United States; ‡ Physics of Complex Fluids − MESA+institute for Nanotechnology, University of Twente, PO Box 217, 7500 AE Enschede, The Netherlands; § Fritz Haber Institute of the Max Planck Society, Faradayweg 4-6, D-14195 Berlin, Germany; ∥ Department of Chemical Sciences, The University of Auckland, Auckland 1142, New Zealand; ⊥ Advanced Light Source, Lawrence Berkeley National Laboratory, Berkeley, California 94720, United States

## Abstract

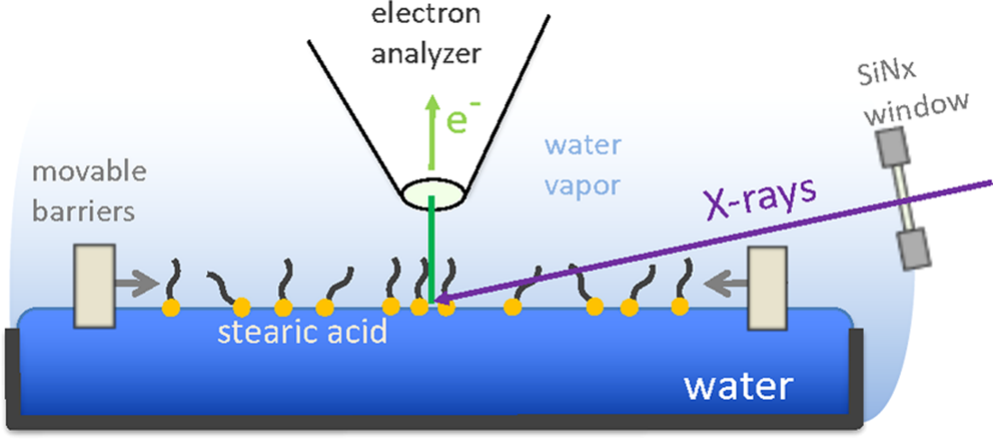

We present a combined Langmuir–Pockels trough and ambient pressure X-ray photoelectron spectroscopy (APXPS) study of the compression of stearic acid surfactant layers on neat water. Changes in the packing density of the molecules are directly determined from C 1s and O 1s APXPS data. The experimental data are fit with a 2D model for the stearic acid coverage. Based on the results of these proof-of-principle experiments, we discuss the remaining challenges that need to be overcome for future investigations of the role of surfactants in heterogeneous chemical reactions at liquid–vapor interfaces in combined Langmuir–Pockels trough and APXPS measurements.

## Introduction

**Figure 1:**
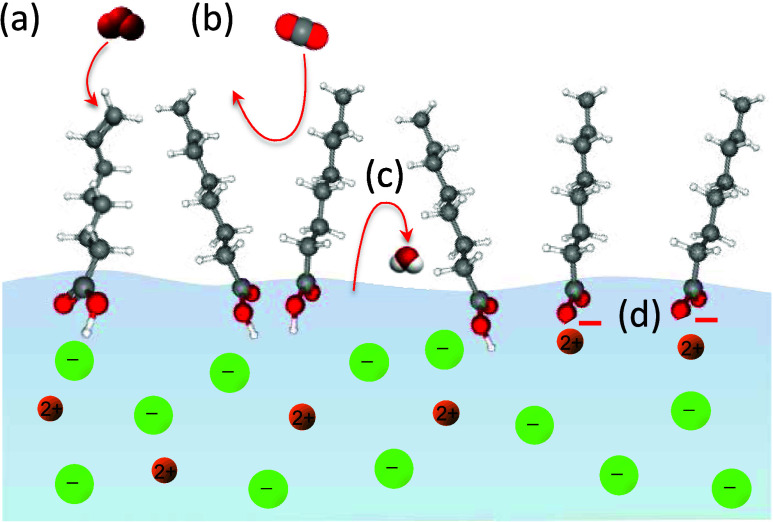
Schematic representation of possible effects of surfactants on heterogeneous reactions at the liquid–vapor interfaces. (a) Direct reaction of gas-phase species with surfactants, for example, ozone with the C=C bond in 7-octenoic acid. (b) Reduced gas molecule uptake at the liquid–vapor interface due to scattering by surfactants. (c) Reduced desorption of molecules from the liquid–vapor interface: here the reduction of the evaporation rate of water by the presence of an octanoic acid layer at the interface. (d) Modification of the propensity of ions in solution through specific interactions between the hydrophilic group of the surfactant with ionic species, for example, the negatively charged −COO^–^ group of octanoate with positively charged ions in solution.

Aqueous solution–vapor interfaces are ubiquitous in nature and govern many important processes, such as the uptake and release of trace gases by aerosol droplets and the sequestration of carbon dioxide by the oceans.^1,2^ Under realistic environmental conditions, the interface is partially or completely covered by hydrophobic or amphiphilic molecules originating from the solution’s bulk or absorbed from the surrounding atmosphere. These mostly carbonaceous surfactants can potentially have a strong influence on the interface chemistry, e.g., by changing the gas transport between the bulk liquid and the atmosphere, by altering the propensity of ions for the interface through electrostatic interactions, or by directly being involved in the heterogeneous reaction (see Figure 1).

Experiments on the influence of long-chained alcohol surfactant layers on the evaporation coefficient of neat water have shown that an increase in the carbon chain length by four carbons can decrease the evaporation coefficient by several orders of magnitude (Figure 1c).^3^ Similar effects are also expected in the opposite gas diffusion direction, i.e., for the migration of vapor molecules through a surfactant layer toward the interface (Figure 1b). On the other hand, ion-scattering experiments demonstrated specific chemical effects of the surfactants on interfacial reactions, specifically that the uptake of gas molecules at a surfactant-covered interface is governed by the chemical nature of both gas molecules and interface.^4^

Surfactant layers can also affect the interface properties through a direct chemical interaction with the constituents of the solution. The presence of a surfactant may, for instance, change the propensity of certain ions for the interface^5^ (see Figure 1d), which in turn can lead to changes in the reaction rates between trace gas molecules and solvated ions. Another possible influence of surfactants concerns their direct reaction with trace gases, such as a reaction between carbonaceous surfactants and strong oxidizers (O_3_, OH^•^, Figure 1a), which can lead to a partial or complete removal of the surfactant layer and an increase in the transport of molecules to or from the liquid–vapor interface. These and other processes, as well as the properties of the liquid–vapor interface, are potentially influenced by the presence and specific chemical nature of the surfactants. For a basic understanding of these phenomena, interface-sensitive studies of model systems with well-controlled chemistry and surfactant coverage are important.

A Langmuir–Pockels trough is an excellent device to prepare liquid–vapor interfaces with adjustable surfactant coverage and chemistry.^6−8^ The basic setup consists of a shallow trough with hydrophobic walls that is filled with the aqueous solution of interest to a level above the height of the trough walls. At the beginning of an experiment, a known quantity of a poorly soluble surfactant is deposited onto the surface of the solution. Two movable barriers are positioned on top of the trough, partially submerged in the water. These barriers are used to define and decrease the surface area available to the surfactant film. The amount of surfactant between the barriers at the beginning of the experiment is well below that necessary to form a closed monolayer. Using the barriers, the surfactant layer can then be compressed, whereby the nominal area available to each surfactant molecule (*i*.*e*., the mean molecular area, MMA) is calculated from the known number of surfactant molecules that were initially deposited and the geometrical area that is enclosed between the barriers and trough walls.

Changes in the packing and the structure of the surfactant layer in a Langmuir–Pockels trough are traditionally monitored through measurements of the surface pressure Π as a function of the MMA, with Π = γ_o_–γ, where γ_o_ is the surface tension of the pure subphase (in most cases water or an aqueous solution) and γ is the surface tension of the subphase with the monolayer.^9,10^ Surface pressure measurements are conducted using, e.g., a microbalance that measures the force acting on a plate or wire that is partly immersed in the liquid. Surface pressure vs MMA curves show characteristic regions that are, in the order of increasing compression, indicative of isolated surfactant molecules (a so-called 2D gas), weak interactions between neighboring molecules, a fully compressed layer, and finally the collapse of the fully compressed monolayer at a critical MMA, above which surfactant molecules can form multiple layers or be driven into the bulk of the solution, where they can form micelles.

The relatively straightforward nature of a Langmuir–Pockels trough setup lends itself to a combination with surface-sensitive methods, most prominently reflection absorption infrared spectroscopy (RAIRS),^11,12^ which provides information on the chemical state and the orientation of surfactant molecules at the solution–vapor interface. Among other methods that have been combined with Langmuir–Pockels troughs is grazing-incidence small-angle X-ray scattering (GISAX),^13−15^ which reveals the structural properties of the film at different stages of compression. An exhaustive review of surface-sensitive methods that have been used to characterize surfactants at the solution–vapor interface can be found in refs (8,16). One of the most popular surface-sensitive characterization methods, X-ray photoelectron spectroscopy (XPS), has been widely used to study surfactant layers after they have been transferred onto solid substrates using the Langmuir–Blodgett and other preparation methods^17,18^ but to the best of our knowledge has not yet been applied to investigations of surfactants at the solution–vapor interface.

XPS provides information on the elemental composition and the chemical nature of the species at the interface (e.g., functional groups) and is thus ideally suited for investigations of heterogeneous chemical reactions involving the gas phase, surfactants at the interface, as well as solvent and solute molecules in the solution (see Figure 1). For investigations of aqueous solutions, the minimum background pressure is given by the equilibrium vapor pressure of water, which is about 25 mbar at room temperature. These experiments thus require the use of ambient pressure XPS (APXPS), which is able to tolerate liquid samples at elevated pressures.^19,20^ We here combine a Langmuir–Pockels trough setup with an ambient pressure XPS experiment to monitor a stearic acid (C_17_H_34_COOH) surfactant layer at different stages of compression, where the C 1s -to- O 1s peak intensity ratio reports on the average thickness and packing density of the surfactant layer.

The experimental approach and proof-of-principle investigations presented in this paper lay the groundwork for future studies of the surface chemistry of surfactants as a function of their packing density, solution pH and ion concentration, and gas-phase composition using APXPS under realistic conditions of water vapor pressure and trace gas concentration. These kinds of studies will provide fundamental insights into the reaction mechanisms at liquid–vapor interfaces with high chemical and interface sensitivity and will provide input for theoretical models of heterogeneous processes in the environment and atmosphere.

## Methods

**Figure 2:**
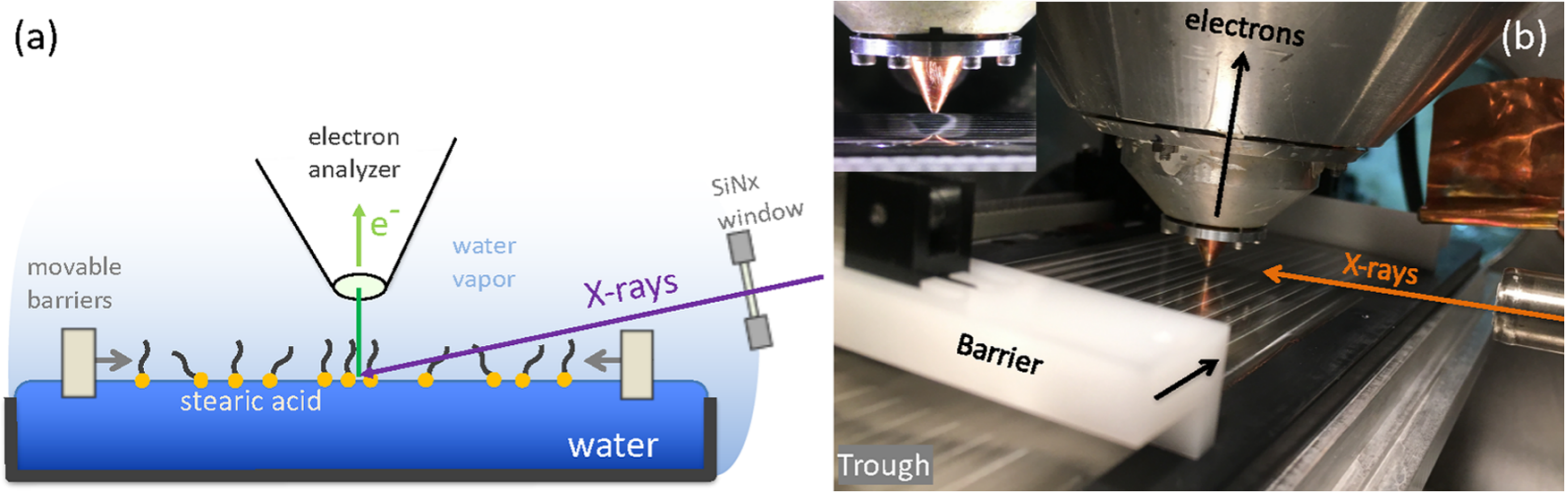
Principal layout of the experiment. (a) X-rays are incident at the liquid surface under a grazing angle of 3°. Photoelectrons and water vapor escape through the entrance aperture (0.2 × 0.4 mm^2^) of the ambient pressure photoelectron spectrometer, which is placed ∼0.5 mm away from the liquid surface to reduce scattering of electrons by gas molecules. The background pressure in the chamber at the beginning of the experiments is ∼23 mbar, which is the equilibrium vapor pressure of water at 20 °C. The distance between the SiNx window and the X-ray spot on the sample is 70 mm. (b) Photograph of the interior of the vacuum chamber. The inset shows a close-up of the entrance aperture of the electrostatic lens system above the solution surface.

A schematic of the experiment is shown in Figure 2. A modified Langmuir–Pockels trough (Kibron Langmuir Trough XS (SS/PTFE)) is placed inside a custom-designed vacuum chamber, which is connected to beamline 11.0.2 at the Advanced Light Source, Berkeley, CA.^21^ The motor controlling the barriers is situated outside the vacuum chamber, connected through a mechanical feedthrough. The trough inner dimensions are 60 mm × 230 mm, with a depth of 1.5 mm. Vertically polarized X-rays are incident at a 3° angle relative to the water surface. The footprint of the X-ray spot on the water surface was determined to be <0.2 mm wide and >5 mm long using a phosphorescent target. Photoelectrons are detected under normal emission, i.e., with an APXPS electron analyzer (Phoibos 150 NAP, SPECS Surface Nano Analysis GmbH, Berlin) perpendicular to the water surface.

APXPS experiments were performed on both pristine and surfactant-covered water surfaces. At the beginning of each experiment, the trough was filled with ∼45 mL of ultrapure water (18.2 MΩcm, Millipore) that was degassed in several freeze–pump–thaw cycles in an external vacuum chamber. Additionally, an extra water reservoir was included in the vacuum chamber to mitigate the excessive evaporation from the trough. The Langmuir–Pockels trough chamber was then evacuated to a pressure of about 40 mbar using a roughing pump. At this pressure, the chamber was isolated from the pump by closing the valve between the chamber and roughing pump, and the remaining air was pumped out through the small entrance aperture (0.2 × 0.4 mm^2^) of the electron spectrometer using the pumps of its differential pumping stages, reaching a pressure of ∼23 mbar after about an hour, which is the equilibrium water vapor pressure at 20 °C, thus indicating that the chamber atmosphere consists overwhelmingly of water vapor. During this time, most of the dissolved air that had entered the water during the filling of the trough was removed, indicated by the disappearance of any visible bubbles in the trough. The pressure in the chamber gradually decreased over the course of the experiments due to evaporative cooling of the water in the trough down to about 18 mbar after several hours, which corresponds to a water temperature of 17 °C.

For the proof-of-principle experiments, the compression of stearic acid on water was chosen as the test system since there is a large body of literature on this molecule,^12,22−24^ to which the present APXPS results can be compared. Stearic acid solutions were prepared by dissolving the pure solid (purity >99%, Merck Millipore) in either toluene or chloroform at a concentration of 1.4 × 10^–3^ mol/L. A small amount of the solution (35 or 45 μL) was carefully deposited onto the water surface before the compression measurements, which required venting and re-evacuation of the chamber according to the procedure described above. The surfactant layer was compressed by moving one barrier and leaving the other in a fixed position. The surface area between the barriers was 8.8 × 10^3^ mm^2^ at the beginning and 4.1 × 10^3^ mm^2^ at the end of the compression.

The C 1s and O 1s core level spectra were recorded to monitor the surface composition as a function of the MMA (determined from the barrier positions), with the adjustable barrier moving at a constant speed of 1.7 mm/min. During each experiment, the barrier traveled 79 mm; i.e., one compression took about 46 min. The incident photon energy was 1300 eV for both C 1s and O 1s spectra, i.e., the kinetic energy of the O 1s and C 1s photoelectrons is about 760 and 1010 eV, respectively, a difference that needs to be taken into account in the quantitative analysis of the C/O ratio, as described further below. The acquisition time for each C 1s and O 1s spectrum was about 90 s, i.e., each spectrum averages over a change in barrier position of about 2.5 mm. A total of 15 pairs of the C 1s and O 1s spectra were recorded during one compression experiment. The combined electron energy analyzer and beamline resolution were better than 0.7 eV.

## Results

**Figure 3:**
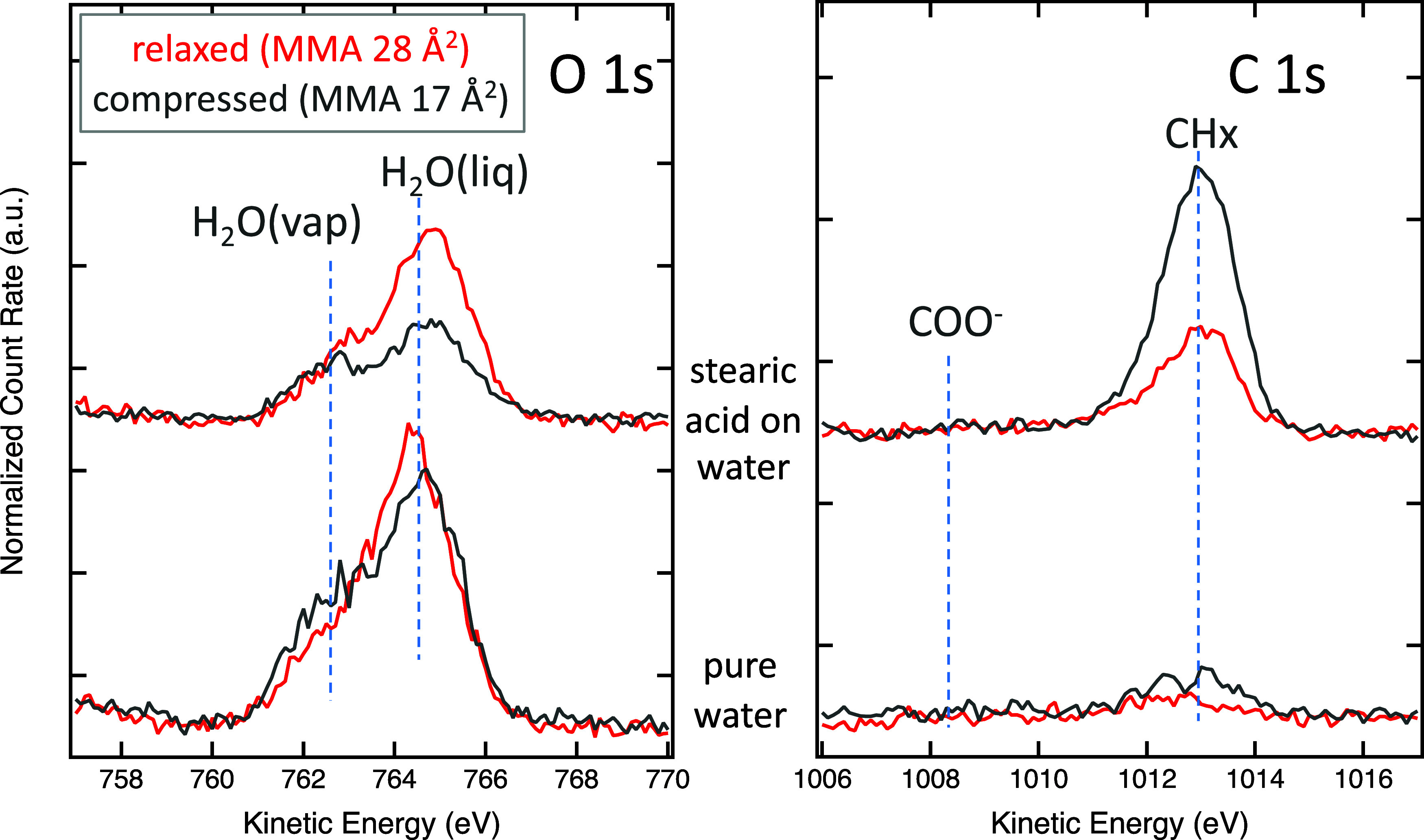
O 1s and C 1s APXPS spectra taken from pure water (bottom row) and a stearic acid layer on water (top row) for two different compression stages. For visual representation, all spectra are normalized to 1 in the high KE region above the photoemission peaks. The average MMAs for the stearic acid spectra are 28 Å^2^ (relaxed) and 17 Å^2^ (compressed). Since the MMA values have no meaning for the nominally clean water measurements, the XPS spectra are taken there at similar barrier positions for a comparison with the stearic acid measurements. The incident photon energy is 1300 eV in all of the spectra.

The upper lines in Figure 3 show the C 1s and O 1s photoelectron spectra of stearic acid on water during a compression experiment at low (red) and high (gray) compressions. The O 1s spectra exhibit two peaks due to the gas phase and liquid water. The C 1s spectra are dominated by the CH_*x*_ peak, which has a nominal binding energy of 285 eV.^25^ The expected peak position of the COO^–^ group (shifted by −4.5 eV relative to CH_*x*_)^26^ is indicated, but this peak is too weak to be identified with certainty, owing to the low abundance (1 in 18) of acid group carbons compared to carbons in the aliphatic tail. The intensity of the acid group C 1s peak is additionally decreased by the scattering of photoelectrons by the aliphatic tail of the molecule since the acid group is located at the solution–vapor interface.

The lower lines in Figure 3 show the C 1s and O 1s spectra of a nominally clean water surface. The O 1s data reveal the expected peaks due to water vapor and liquid water, while the C 1s data indicate that–despite careful preparation of the pure water sample–there is still carbonaceous material present. The C 1s spectra taken with the barriers at their closest position show a slight increase in C 1s intensity compared to the relaxed case, as expected for surface-active contamination species. The signal-to-noise ratio in the C 1s spectra for nominally pure water is too low to deduce the chemical nature of the contamination, but the main intensity is found at the characteristic position for CH_*x*_.

**Figure 4:**
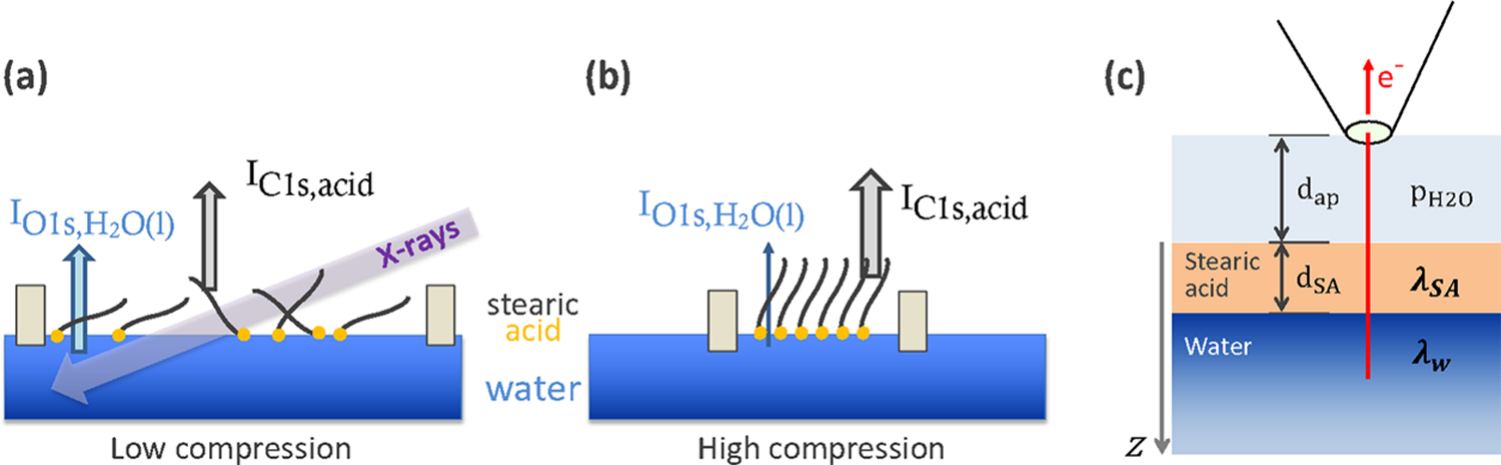
Change of the C 1s signal from the surfactant layer and the O 1s signal of H_2_O(l) when going from (a) low coverage to (b) high coverage. (c) For the quantification of the effective surfactant layer thickness *d*_SA_, the inelastic mean free path of the electrons in water (λ_W_) and in stearic acid (λ_SA_) has to be taken into account. In addition, the attenuation of the electrons by water vapor at a pressure of p_H2O_ over the distance *d*_ap_ needs to be considered since the kinetic energy of the O 1s and C 1s photoelectrons (and thus their attenuation) differs.

From the measured C 1s and O 1s peak areas in the spectra like the ones shown in Figure 3, one can quantify the coverage of water by stearic acid as a function of compression. For low coverages of stearic acid on water (Figure 4a), i.e., at low compression, the O 1s signal from water is less attenuated by stearic acid, and there are fewer stearic acid molecules per unit area at the water surface, resulting in a low C 1s/O 1s peak area ratio. This ratio increases as the stearic acid layer is compressed (Figure 4b). This behavior is observed in the XPS data for the stearic acid layer shown in Figure 3, upper row. The peak area of H_2_O(l) decreases with increasing compression of the stearic acid layer, as expected from the higher degree of electron scattering at higher compression. At the same time, the C 1s signal from the surfactant layer increases due to the higher density of the film with increasing compression.

A quantitative comparison of the C 1s and O 1s peak areas allows an estimate of the stearic acid coverage using a 2D model for the surfactant film on water, as shown in Figure 4c. This simplified model assumes homogeneous coverage (i.e., no islands) and thus results in an average value for the film thickness *d*_SA_. For the quantitative analysis of the C/O ratio as a function of the MMA, the measured C 1s and O 1s peak areas need to be normalized by their respective photoemission cross-section^27^ and inelastic scattering by the water molecules in the gas phase.^28^ Since the incident photon energy and beamline settings are the same (1300 eV) for both core levels, the photon flux does not factor into the determination of the C/O ratio. Likewise, the asymmetry parameters in the photoemission cross-section cancel out since both core levels are of *s*-type and are measured in the same geometry and X-ray polarization.

The attenuation of the photoelectrons by the water gas phase is governed by
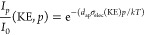
1with *I*_*p*_ as the measured photoelectron intensity at pressure *p*, *I*_0_ the intensity in vacuum, *d*_ap_ the distance between the spectrometer lens aperture and the surface, σ_elec_ the total electron scattering cross-section of water^28^ at the given kinetic energy KE, *T* the temperature of the gas phase, and *k* the Boltzmann constant. For a pressure of 21 mbar and *d*_ap_ = 0.5 mm, the attenuation ratios for KEs of 760 and 1010 eV are 0.004 and 0.011, respectively, i.e., the photoelectron intensity decreases by a factor of ∼250 in the case of O 1s and ∼90 in the case of C 1s with respect to a hypothetical measurement under vacuum. This calculation assumes that the pressure decreases abruptly by several orders of magnitude right behind the entrance plane of the differentially pumped aperture, which is a reasonable assumption based on previous calculations of differentially pumped entrance apertures of APXPS lens systems and is supported by a finite element simulation (*Comsol Multiphysics*).^29^Table 1 summarizes the factors that enter into the normalization of the C 1s and O 1s peak areas. The good agreement of the theoretical and experimentally determined (from the CO_2_ gas-phase measurements at ∼0.2 mbar) cross-sections demonstrates that differences in the electrostatic lens transmission at 760 and 1010 eV are negligible.

**Table 1:** Parameters for the Normalization of the O 1s and C 1s Intensities for an Incident Photon Energy of 1300 eV^a^

	KE (eV)	photoemission cross-section^27^ σ_PE_ (Mb)	C 1s/O 1s cross-section ratio (calculated)	C 1s/O 1s cross-section ratio from CO_2_(*g*)	total electron scattering cross-section^28^ of water σ_elec_ (10^–20^ m^2^)	attenuation factor *I*_p_/*I*_0_ from eq 1	C 1s/O 1s sensitivity factor
O 1s	760	0.05	0.4	0.45	2.0	0.004	1.17
C 1s	1010	0.02			1.64	0.011	

a The value for the gas-phase attenuation factor *I*_p_/*I*_0_ was calculated by using a water vapor pressure of 21 mbar and a sample–aperture distance of 0.5 mm using eq 1. The experimentally observed O 1s/C 1s ratio was obtained from CO_2_(*g*) measurements at p_CO_2__ ∼ 0.65 mbar, where gas-phase scattering of photoelectrons is negligible compared to that at 21 mbar. The C 1s/O 1s sensitivity factor was calculated from the average of the theoretical and experimentally determined cross-section ratio (0.425) and the gas-phase attenuation factors.

**Figure 5:**
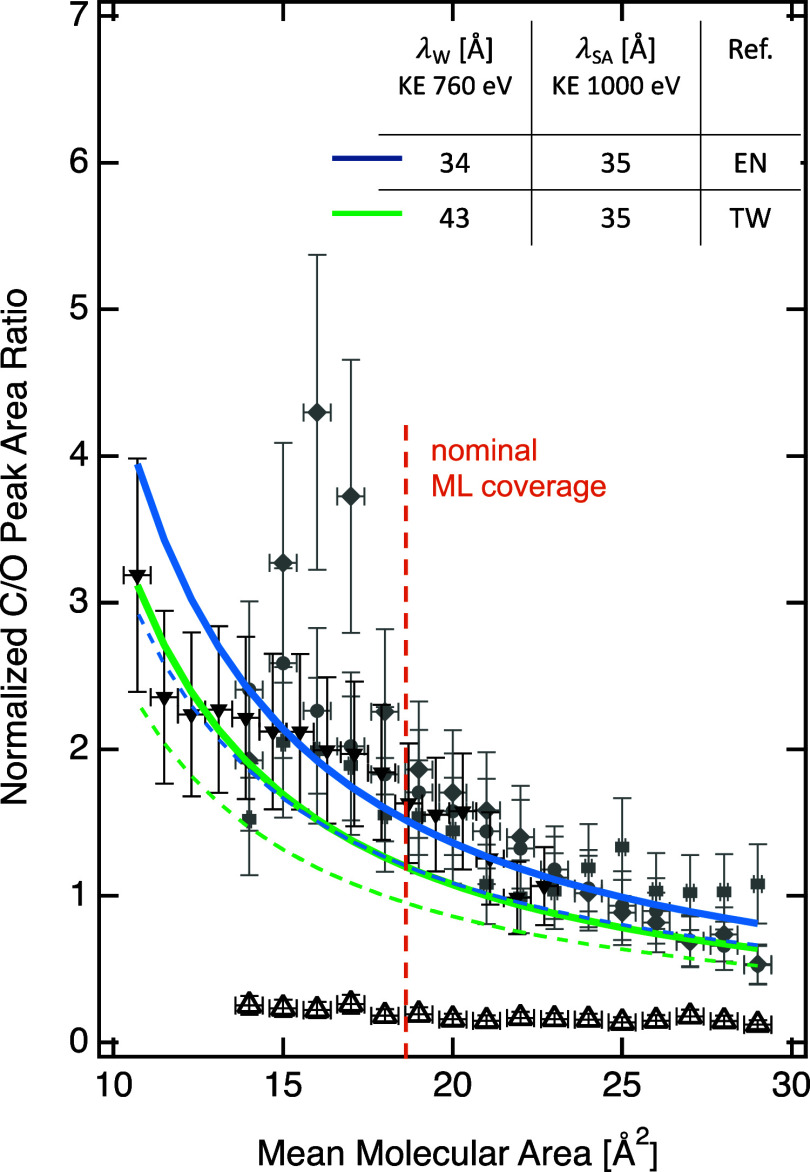
Normalized C/O peak area ratios for 4 different compression isotherms of stearic acid on neat water. Full gray symbols represent 3 compressions with the same starting coverage of stearic acid and full black symbols a compression with higher starting coverage. Open triangles are compressions of a nominally clean water surface. The solid lines are expected C/O ratios based on the model in eqs 2–4 using the values for the inelastic mean free path from Emfietzoglou and Nikjoo (EN)^32^ and the effective attenuation length from Thürmer et al. (TW)^33^ for a specific molecular volume of 590 Å^3^ per stearic acid molecule. The dashed lines are for a molecular volume of 505 Å^3^. See the text for details.

Figure 5 shows the normalized C/O intensity ratios as a function of MMA for four different compression isotherm experiments of stearic acid on neat water. Solid gray symbols represent three isotherms that were prepared with a starting stearic acid coverage corresponding to an MMA of 29 Å^2^, while the solid black triangles are the results of an experiment with a higher starting coverage of stearic acid (MMA 23 Å^2^). For comparison, the open triangles show the data for nominally clean water. The vertical error bars represent uncertainties in the normalization of the C 1s and O 1s cross-sections and the degree of electron scattering by gas-phase molecules. Horizontal error bars account for the fact that the O 1s and C 1s spectra were taken sequentially, and thus each data point is an average over an MMA interval of ∼0.8 Å^2^. The vertical dashed orange line in Figure 5 indicates the literature value for the MMA of the compressed stearic acid monolayer (ML).^12^ The compression isotherm C/O ratios in Figure 5 show good agreement between the four different experiments below the nominal monolayer compression (i.e., at high MMAs), while there are noticeable differences above monolayer compression (i.e., at low MMAs). This variability can arise from differences in the accommodation of the excess monolayer packing in the different samples by, e.g., submersion of parts of the stearic acid layer into the aqueous subphase or the formation of multilayers.

Based on a 2D layer model for the coverage of stearic acid on water (see Figure 4c), we now calculate the expected C/O ratio as a function of MMA. We assume that the stearic acid film has a homogeneous thickness across the whole area between the barriers of the Langmuir–Pockels trough. The specific volume for a single stearic acid molecule (v_SA_) can be calculated from the unit cell of crystallized stearic acid, which has a volume of 1180 Å^3^ and contains two stearic acid molecules;^30^ hence, v_SA_ = 590 Å^3^. An alternative way is to calculate the specific volume from the molar mass (284.5 g/mol) and mass density^31^ (0.94 g/cm^3^), which yields v_SA_ = 504 Å^3^ per molecule. From the specific volume of a stearic acid molecule, one can obtain the density of C atoms in stearic acid, which is 30 nm^–3^ when calculated from the unit cell dimensions and 36 nm^–3^ when calculated from the molar mass and mass density. The average of these values is equal to that for water (33 nm^–3^), calculated from the molar mass and mass density, and thus, the relative atomic density of C in stearic acid and O in water cancel out in the following calculations.

For an estimate of the expected C/O ratio, the C 1s intensity is calculated according to (see Figure 4c)
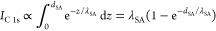
2by integrating over the stearic acid film thickness, starting at the water vapor–stearic acid film interface. The O 1s intensity of water in the subphase is calculated by integrating from the stearic acid film–water interface into the bulk and taking into account the attenuation of the liquid water O 1s intensity by the presence of the stearic acid film
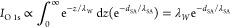
3with λ_SA_ and λ_W_ as the inelastic mean free paths of stearic acid and water at their kinetic energy of 1000 and 760 eV, respectively. Accounting for the effective stearic acid film thickness *d*_SA_ = *v*_SA_/MMA, the calculated peak area ratio is
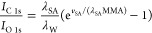
4The solid lines in Figure 5 are calculated C/O ratios by using eq 4 for different values of the inelastic mean free path in water (λ_W_) and stearic acid (λ_SA_), assuming *v*_SA_ = 590 Å^3^. The blue and green lines are based on calculated inelastic mean free paths (IMFP)^32^ and experimentally determined effective attenuation lengths^33^ for liquid water, respectively. IMFP values for stearic acid were calculated using the NIST Standard Reference Database 71,^34^ assuming a band gap of 7.6 eV^35^ and a density of stearic acid of 0.94 g/cm^3^. This resulted in an IMFP of 3.6 nm (TPP-2 M equation)^36^ and 3.4 nm (G-1 equation);^37^ in the calculations of the theoretical C/O ratio, we have used the average value of 3.5 nm. For comparison, the dashed lines in Figure 5 are calculated C/O ratios using *v*_SA_ = 505 Å^3^, based on the bulk density and molar mass of stearic acid. The best fit with the experimental data is achieved using λ_W_(760 eV KE) = 3.4 nm, λ_SA_(1000 eV KE) = 3.5 nm, and v_SA_ = 590 Å^3^.

The discussion above demonstrates that several factors and assumptions have a strong influence on the interpretation of the measured C/O ratios and that the uncertainties in these numbers need to be addressed for a more reliable, quantitative description of surfactant layers on aqueous interfaces. In the following, challenges and opportunities for the investigation of aqueous–vapor interfaces in a combined Langmuir–Pockels trough/APXPS setup are discussed.

## Discussion

The biggest challenge for the investigation of static liquid–vapor interfaces using APXPS and other surface-sensitive methods is the preparation of clean interfaces with a well-defined chemical composition. As in any other surface-sensitive experiment, the adsorption of contamination either from the bulk or from the surrounding vapor phase or vacuum to the interface needs to be avoided or slowed to a significant degree. Traditional surface science experiments at solid–vacuum interfaces rely on an array of sample preparation strategies that remove unwanted contamination from the interface such as sputtering and annealing. Contamination levels in these kinds of experiments are further kept at bay by measuring at very low background pressures, in the 10^–9^ mbar range or below.

These strategies are clearly not available for the investigation of aqueous solution–vapor interfaces under environmentally relevant conditions,^20^ where the solution vapor pressure is in the range of a tenth to tens of millibar. The equivalent of well-controlled surface science-type studies for aqueous solutions is the use of liquid microjets,^38^ which are injected at speeds of >10 m/s through a small orifice (typical diameters are 10 to 50 μm) into a vacuum chamber. After traversing the vacuum chamber, the liquid is frozen out using a liquid nitrogen trap, keeping the background chamber pressure in the sub 10^–3^ mbar range. Contamination from the vacuum side is suppressed since the laminar portion of the jet is measured within ∼1 mm distance after injection of the liquid into the measurement chamber, i.e., within less than 1 ms after the surface is in contact with the gas phase. This allows the performance of XPS experiments in the absence of any measurable contamination. Liquid jet experiments in general do not allow, with a few exceptions,^39^ the investigation of gas–liquid reactions due to the short contact time between the liquid and the vapor before the measurement; for these types of investigations, static liquid–vapor interfaces are more suitable.

**Figure 6:**
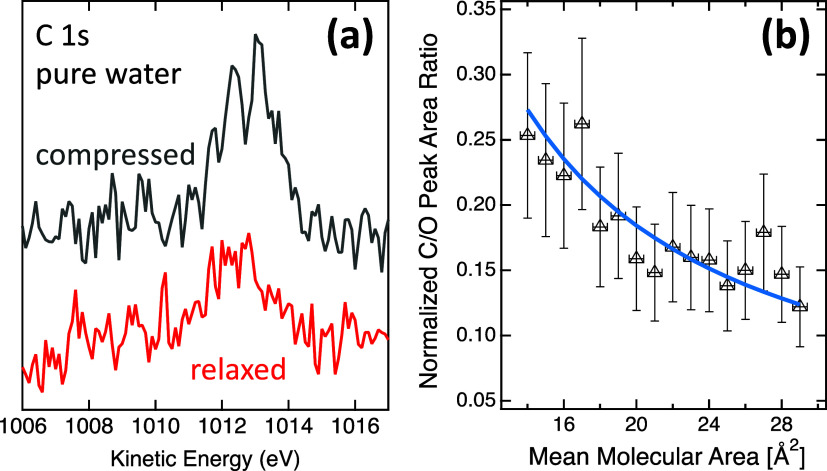
(a) Same C 1s spectra of nominally clean water as shown in Figure 3, here enlarged. (b) Normalized C/O peak area ratio as a function of nominal MMA for clean water. This is the same data set as also shown in Figure 5. The blue line is a fit for the thickness of the carbonaceous layer, using the IMFP values for the best fit in the case of stearic acid, i.e., λ_W_(760 eV KE) = 3.4 nm and λ_SA_(1000 eV KE) = 3.5 nm, yielding a value of 112 Å^3^/MMA.

The difficulty in preparing clean static liquid–vapor interfaces is demonstrated by the C 1s spectra for nominally clean water, shown in Figure 3. These spectra are again enlarged in Figure 6a. With the barriers at a large separation, there is clearly some carbonaceous material at the liquid–vapor interface. The peak intensity of this species (or mix of species) is found at the same KE as that of the aliphatic tail of stearic acid. When the barriers are closed, the amount of carbonaceous material increases, which demonstrates that this is indeed a surfactant-type contamination. Figure 6b shows the C/O peak area ratio (already displayed in Figure 5, and here enlarged) as a function of nominal MMA (referenced to stearic acid). The blue solid line is a fit of the data by eq 4 using the IMFP values for the best fit in the case of stearic acid, i.e., λ_W_(760 eV KE) = 3.4 nm and λ_SA_(1000 eV KE) = 3.5 nm, resulting in a value for the thickness of the carbonaceous contamination of 112 Å^3^/MMA. Thus, the nominal thickness of the contamination at the beginning of the experiment is 3.8 Å and at the end of the compression 7.5 Å, i.e., double the thickness as at the beginning when the available surface area is decreased by a factor of about 2.

The neat water sample was prepared with great care from deionized (Millipore) water, which was subsequently degassed in several freeze–pump–thaw cycles. Nevertheless, these procedures do not completely remove surface-bound organics from water, which are also in equilibrium with dissolved species in the bulk of the solution. In addition, contamination can adsorb on the sample during the transfer of the water (using a clean pipet) between the degassing vessel and the Langmuir–Pockels trough inside the APXPS experimental cell. Contamination can also migrate from the interior walls of the chamber to the water surface, a process that is aided by the reduced background pressure in the chamber (extending the mean free path of gas-phase molecules) and the fact that the sample surface is the coldest part of the chamber due to evaporative cooling. It should be noted that the degree of contamination is, despite all of these challenges, in line with what is expected for any other sample that is transferred from air into a vacuum chamber. A contamination level that is akin to one or two monolayers of carbon is hardly surprising. The challenge in the case of static liquid–vapor interfaces is that post-transfer in-vacuum preparation methods, such as sputtering and annealing, are not available. It is thus necessary to develop chemical or physical routes for the in situ cleaning of the liquid–vapor interfaces.

We finish the discussion by pointing out that the preparation of surfactant films with controlled chemical composition and coverage can be of value for the determination of the inelastic mean free path of electrons in water and aqueous solution, a topic that is still under debate.^40^ This requires measurement of the surface tension of the solution inside a vacuum chamber to reliably control the compression of the surfactant film. The inelastic mean free path of electrons in the surfactant film can be determined experimentally on films transferred onto solid substrates using the Langmuir–Blodgett technique by comparing the substrate photoelectron intensity with and without the surfactant film, where the density and homogeneity of the transferred surfactant layer can be independently verified using imaging techniques, such as scanning force microscopy.^41^ In this manner, λ_W_ can be determined from the measured C 1s and O 1s intensities using eq 4.

## Conclusions

We demonstrated the feasibility of combined Langmuir–Pockels trough/APXPS investigations in proof-of-principle experiments with the example of the compression of stearic acid layers on water. The measured C/O peak intensity ratios agree with calculated intensity ratios based on a 2D layer model. The experiments also demonstrate the need for the development of suitable in situ surface cleaning methods for liquid–vapor interfaces. We postulate that the general approach shown in the present experiments is the basis for future investigations of heterogeneous chemical reactions with relevance to atmospheric and environmental science.
